# Surveillance programmes for hepatocellular carcinoma: evident-based medicine is not evidence-based medicine

**DOI:** 10.1111/j.1478-3231.2008.01876.x

**Published:** 2009-01

**Authors:** Alain Braillon

**Affiliations:** Public Health, University Hospitals AmiensFrance

To the Editor:

The paper concerning surveillance programmes for hepatocellular carcinoma (HCC) in the journal needs one clarification (1).

Indeed, survival is lower when the diagnosis is made late than soon, and when the patient is older. However, this does not demonstrate the usefulness of surveillance. Several biases exist, the main being the lead-time bias. Again, here is an example concerning HCC. In a recent study, screened patients died 18 months earlier than non-screened ones; nevertheless, the authors concluded that screening improved survival (2)! Numerous studies have investigated screening; all were negative or inconclusive (3, 4).

Moreover, at present, no randomized-controlled trials are available to choose potentially curative treatments for small HCC: transplantation, resection and local ablation.
